# Maternal and newborn health priority setting partnership in rural Uganda in association with the James Lind Alliance: a study protocol

**DOI:** 10.1186/s40900-020-00231-4

**Published:** 2020-09-22

**Authors:** James Ditai, Monicah Nakyazze, Deborah Andrinar Namutebi, Proscovia Auma, Martin Chebet, Cynthia Nalumansi, Grace Martha Nabulo, Kenneth Mugabe, Toto Anne Gronlund, Anthony Mbonye, Andrew D. Weeks

**Affiliations:** 1 Sanyu Research Unit, Department of Women’s and Children’s Health, University of Liverpool, Liverpool Health Partners, Crown Street, Liverpool, L8 7SS UK; 2 Sanyu Africa Research Institute (SAfRI), Mbale Regional Referral Hospital, Pallisa road, Mbale, Uganda; 3 Busitema University Faculty of Health Sciences, Mbale, Uganda; 4 Mbale Regional Referral Hospital, Mbale, Uganda; 5 Step radio FM station, Nkokonjeru road, Mbale, Uganda; 6 Busiu HCIV, Mbale District local government, Tororo Road, Mbale, Uganda; 7 The James Lind Alliance, Trials and Studies Coordinating Centre, National Institute for Health Research Evaluation, University of Southampton, Alpha House, Enterprise Road, Southampton, Southampton, SO16 7NS UK; 8 School of Public Health, Makerere University College of Health Sciences, Kampala, Uganda; 9 Sanyu Research Unit, Department of Women’s and Children’s Health, University of Liverpool, Liverpool Health Partners, Crown Street, Liverpool, L8 7SS UK

**Keywords:** Maternal, Newborn, Health, Research priorities, James Lind Alliance

## Abstract

**Background:**

Maternal and newborn deaths and ill health are relatively common in low income countries, but can adequately be addressed through locally, collaboratively designed, and responsive research. This has the potential to enable the affected women, their families and health workers themselves to explore ‘why maternal and newborn adverse outcomes continue to occur. The objectives of the study include;

1. To work with seldom heard groups of mothers, their families, and health workers to identify unanswered research questions for maternal and newborn health in villages and health facilities in rural Uganda
2. To establish locally responsive research questions for maternal and newborn health that could be prioritised together with the public in Uganda
3. To support the case for locally responsive research in maternal and newborn health by the ministry of health, academic researchers and funding bodies in Uganda.

**Methods:**

The present study will follow the James Lind Alliance (JLA) Priority Setting Partnership (PSP) methodology. The project was initiated by an academic research group and will be managed by a research team at the Sanyu Africa Research Institute on a day to day basis. A steering group with a separate lay mothers’ group and partners’ group (individuals or organisations with interest in maternal and newborn health) will be recruited. The PSP will be initiated by launch meetings, then a face-to-face initial survey for the collection of raw unanswered questions; followed by data collation. A face-to-face interim prioritisation survey will then be performed to choose questions before the three separate final prioritisation workshops.

The PSP will involve many participants from an illiterate, non-internet population in rural eastern Uganda, but all with an interest in strategies to avert maternal and newborn deaths or morbidities in rural eastern Uganda. This includes local rural women, their families, health and social workers, and relevant local groups or organisations.

We will generate a top 10 list of maternal and newborn health research priorities from a group with no prior experience in setting a research agenda in rural eastern Uganda.

**Discussion:**

The current protocol elaborates the JLA methods for application with a new topic and in a new setting translating the JLA principles not just into the local language, but into a rural, vulnerable, illiterate, and non-internet population in Uganda. The face-to-face human interaction is powerful in eliciting what exactly matters to individuals in this particular context as opposed to online surveys.

This will be the first time that mothers and lay public with current or previous experience of maternal or neonatal adverse outcomes will have the opportunity to identify and prioritise research questions that matter to them in Uganda. We will be able to compare how the public would prioritise maternal health research questions over newborn health in this setting.

## Plain English summary

There is increasing recognition for public involvement in research. It ensures that research is relevant to the needs of the end-users. However, currently, research ideas arise mainly from academics and health professionals without the involvement of those who directly experience the problem or conditions. This is especially important in rural low-income settings where there are often huge cultural and economic differences between researchers and the population. We, therefore, aim to work with seldom-heard groups of mothers, their families, social and health workers in rural eastern Uganda. We will identify the unanswered research questions on pregnancy, childbirth, and newborn care that are considered important to the women and their families who receive such care.

## Background

Maternal and newborn health exist together as a public health priority at international, national, regional, and local levels [1]. Stillbirths, neonatal deaths, maternal morbidity, and mortality remain important health issues facing health workers, women, their families, and the community [2].

In Uganda, there are approximately 38 perinatal deaths per 1000 pregnancies (stillbirths and early neonatal deaths), 27 neonatal deaths per 1000 live births, 336 maternal deaths per 100,000 live births but these vary from one area to another [3]. Maternal near-misses, pregnancy-related illnesses, and other potentially devastating consequences after childbirth occur in many women [4].

Poor pregnant women are intensely vulnerable to illness, disability, and even death or any other costs incurred during childbirth. Perinatal mortality is twice in women with no education (45 deaths per 1000 pregnancies) compared to women with more than a secondary education in Uganda [3]. These maternal and newborn health challenges in low-income regions (LIRs), can adequately be addressed through locally, collaboratively designed, and responsive research. This involves working with local women and communities to choose what research is undertaken [5].

However, the health research prioritisation for maternal and newborn health has mostly occurred at the highest international levels and in developed countries, [6]. Where research priority setting has occurred in LIRs regions, like Uganda [7–9], it was set by researchers, academicians, policymakers, and high-level professionals, who do not live the life of those affected.

There is a considerable variation in approaches, tools, and methods used for setting priorities in health research [10], but the James Lind Alliance (JLA) method has recently emerged as the most rational choice designed for clinicians to work together with their patients or laypeople to generate research questions of direct local relevance and benefit. The JLA method has been in use in the UK since 2004 before its application in Canada and Netherlands and is a major method for setting priorities in health research [11]. It’s designed to highlight research questions that are of direct relevance and potential benefit to patients and the clinicians who treat them.

We are working in association with the James Lind Alliance to set up a maternal and newborn health priority setting partnership in rural eastern Uganda (MNH-UG PSP). The study article aims to present this research prioritization process to identify unanswered questions on pregnancy, childbirth and newborn care considered most important to the women and their families, health workers, and social workers in rural Uganda. We will consider effective ways of applying the James Lind Alliance process in a rural and vulnerable population in Africa. The study will provide substantial new learning for ensuring the voices of seldom heard and complex rural populations in sub-Saharan Africa are heard to form the research agenda.

## Methods

### Context and scope

#### Geographical scope

The PSP will take place in the districts of Mbale and Budaka, a rural setting in eastern Uganda. We will specifically recruit from four health facilities, namely Mbale regional referral hospital at health centre level 6, three community health centres at level 4 and 3, social services centres and the surrounding 10 villages. We will choose a facility in a central area with some urban population, follow compass bearings visually aligned to the main road outlets of Mbale town to map out community health facilities (east, west and south) with a largely rural population. We will map out villages around each facility using the sub-county Maps, from which 2 villages will be selected to participate in the study.

#### Health field

**Fig. 1:**
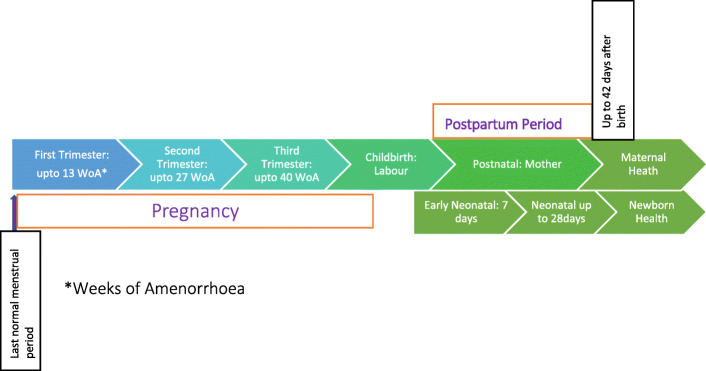
PSP Scope

Preliminary discussions via zoom, email, and one-day meetings were undertaken by the academic research group about the scope of the project. This informed the steering group discussion to have a broad scope, integrating both maternal and newborn health (Fig. 1). Maternal and newborn health are integrated. This scope covers conditions or aspects of care during pregnancy, the intrapartum period, the immediate postpartum period, the neonatal period, and the management of complications that develop during this period [12–14].

#### Research area

This will be global maternal and newborn health.

#### Type of research questions

Though this might vary, we expect the questions to cover aspects of aetiology, diagnosis, prevention, treatment or interventions, care, prognosis, health services, psychosocial, behavioural and social science, economic evaluation, or implementation science.

#### Intended beneficiaries

Many of the people in the prioritisation process will be from an illiterate, non-internet population in rural settings of eastern Uganda. The process is expected to apply to all women, their families, social and health service providers across wider settings in Uganda. It might be extrapolated to a similar population in other low-income regions across sub-Saharan Africa.

#### Target audience for the priorities

We plan to share research questions with the following agencies that have the potential to implement;

1. *Policymakers*.

Ministry of Health, Ministry of Education and sports, Ministry of Gender, labour and social development, Ministry of Science, Technology, and innovation in Uganda; World Health Organisation.

2. *Clinical care organisations*

All clinical care-related questions will be prepared and submitted to the Mbale regional referral hospital, Mbale district health office, Mbale district local government, Budaka District health office, Budaka district local government, and Ministry of Health.

3. *Researchers*

We will prepare a report with all research priorities and share with World Health Organisation, and academic institutions in Uganda namely Busitema University, Makerere University, Mbarara University, Gulu University, Uganda Martyrs University, Uganda Christian University and Lira University. The students or faculty of Master of Public Health, Master of Medicine, Masters of Midwifery and Bachelor in midwifery will be encouraged to implement the research questions as partial requirements for fulfilment of their programmes.

4. *Funders*

We will prepare a study report with a list of top research questions and share with funding and research agenda setting organisations, namely; the Medical Research Council (MRC), National Institute of Health Research (NIHR), Bill and Melinda Gates Foundation, Global Fund, as well as any other major research funding bodies.

#### Timeframe

**Fig. 2:**
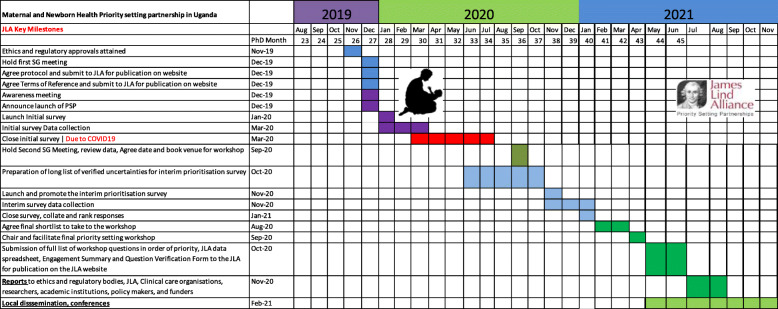
PSP timelines

The PSP will open and last for 12–18 months of implementation. The outbreak of COVID-19 pandemic and the lockdown restrictions in Uganda have subsequently affected our timelines. The study is expected to complete in August 2021 (Fig. 2).

### Framework for research priority setting

The study will follow the James Lind Alliance (JLA) priority setting partnership (PSP) method, well described in a freely available on-line manual [11]. This is a multi-stepped pragmatic method for research priority setting [15, 16]. The JLA PSP brings together patients, carers, and health professionals to identify treatment uncertainties, which become research questions [15–17]. The method considers the opinions of ‘*experts by experience*’. The method is “focussed on being inclusive, transparent, and evidence-based” [11, 16]. The current protocol elaborates these methods for use with a new topic and in a new setting.

### Theoretical framework

The present PSP is underpinned by a socio-ecological model (SEM) [18, 19], a system of classifying the main influences in research priority setting in low-income regions. The model has its foundation in the social choice theory [20], combining individual preferences to reach a collective decision, as well as the utility theory [21], understanding the interest of individuals before talking of the interest of the community. This model has the potential to enable women themselves to explore ‘why maternal and newborn adverse outcomes continue to occur?’ [22]. In this way, it can identify research questions that apply to the local context using a transparent method [15]. We will apply the SEM model whenever selecting participants at each phase of the prioritisation process.

### Initial enquiry

In October 2016, the academic research group (the University of Liverpool and Sanyu Africa Research Institute) contacted JLA via an email expressing interest to set up a PSP for maternal health in Uganda as part of a Ph.D. training proposal. The academic research group made an initial enquiry with JLA to initiate and develop the process for priority setting.

JLA shared the PSP information for guidance including a readiness questionnaire and found us not ready to start due to lack of funding. However, we continued to develop the idea.

Upon confirmation of funding for the Ph.D. training in 2017, the potential for a PSP was discussed again with JLA in November 2017, and a half-day meeting in London in June 2018. The attendees represented the University of Liverpool, University College London, University of Leicester, University of Leicester, the James Lind Alliance, and the Sanyu Africa Research Institute in Uganda. The meeting discussions included the scope, which initially included gynaecological conditions; a discussion of a grant application for the PSP. We later submitted a grant application to MRC, Health systems Research initiative foundation call, which was not funded, with a notification in October 2018.

The academic research group submitted a similar application with the scope of maternal and newborn health as part of the BabyGel trial grant application to the European and Developing Countries Clinical Trials Partnership (EDCTP). This was funded and commenced in February 2019. The Academic research team immediately engaged JLA again via zoom to discuss the readiness to implement the PSP, its implementation modalities for academic training purposes, and agree on the JLA chair.

### Form the leadership and management team

#### Governance and team

The PSP is set up primarily for academic training purposes, with the leadership and management team of four groups, namely; academic research group, steering group, lay mothers’ group, and partners’ group.

The academic research group consists of the academic faculty (supervisors) of the University of Liverpool and Makerere University, the student, and the research team.

This group formed after registration for Ph.D. training with the University of Liverpool as an offsite student based at the Sanyu Africa Research Institute. This is a research institute in rural Uganda that uses research and innovations to improve outcomes for mothers and newborns (www.safri.ac.ug). The institute will serve as the local coordinating organisation for this PSP with the research team.

The research team will consist of research assistants (3 females) led by the student (male). This will form the secretariat responsible for managing the PSP on a day to day basis in Mbale. The research team members will have a background in medical, nursing, midwifery, and or social sciences, basic skills in qualitative research methods, and be fluent in any one local language (Lumasaba, Lugwere, Luganda, Ateso). They will be trained in the PSP and JLA method. They will coordinate, administer, and manage the overall process in Mbale including organising meetings and workshops, collecting, managing, and analysing data.

The steering group includes membership of mothers, carers, health, and social workers with experience or interest in pregnancy, childbirth, and newborn health. This will be a diverse group of 15 members, to be recruited by the academic research group (Additional file 1 describes the characteristics of members). The student purposively identifies persons based on peer knowledge and personal contacts or experience in this particular setting. In this particular PSP, the student made phone calls and physical face to face conversations with 14 potential mother and carer representatives, discussed the project, and invited the 7 for membership. Meanwhile, 10 clinical care representatives were talked to via phone call or face to face and selected 7 health workers to the membership. Each member was sent an appointment letter, meeting invitation letter via email, and a hard copy delivered at the time of the first meeting.

JLA provided an independent chair for the steering group, who set up and signed a contract with the academic research group/ Sanyu Africa Research Institute. The tasks and roles of the steering group will be in line with the JLA guidebook. JLA adviser will chair the group meetings and facilitate the final priority setting workshop in Mbale. The academic research group will keep as observers to the steering group.

The lay mothers’ group is an advisory group of 5 lay mothers to the steering group. It includes mothers with little or no formal education, whose voice is hardly heard of, and living in hard to reach villages of Uganda, with previous experience or adversity of pregnancy, childbirth, and or newborn health as a typical African woman. The purpose of this subgroup is to actively engage these disadvantaged and marginalised group of women whose voice and influence could easily be lost in the steering group.

The mother and clinical representatives to SG with personal contacts approached this kind of mother for participation in the group.

The subgroup has a representative on the steering group, who is a traditional birth attendant and understands multiple local languages in the area. S/he will chair the subgroup meetings and present their views or voices to the Steering group.

The research team will organise a one-day training for the subgroup in a basic introduction to the principles of public involvement and James Lind Alliance. The research team will organise subgroup meetings a week before any steering group meeting throughout the PSP. The research team will take minutes and remain as observers to the subgroup’s meetings. The minutes from the subgroup meeting will be used to ensure their voices are appropriately represented to the steering group.

### Identify and invite partners

Partners’ group will be made up of individuals or organisations with interest in pregnancy, childbirth, and newborn health in rural eastern Uganda (Additional file 1). Their role will be to execute actions decided by the steering group. They will support and promote the PSP, help spread the message about the PSP to their contacts, publicise the PSP surveys to the staff and their clients, encourage their members to take part in the prioritisation processes and disseminate the results.

#### Selection

The student, a research team member will identify 20 individuals and organisations from which 10–15 will be selected and confirmed to get involved in the PSP by the steering group.

##### Initial meetings

The study will be initiated by four separate initial meetings amongst the leadership and management team, occurring on different days. Additional file 2 shows the minutes of the initial meetings held.

### Stakeholders or participants

#### Study population

The present study involves many of the participants from an illiterate, non-internet population in rural eastern Uganda. These include women, their families, health and social workers, and relevant local groups or organisations.

Individuals will be eligible to participate in the surveys and priority setting workshops if they are at least 16 years of age, live in eastern Uganda, and have experience or interest in pregnancy, childbirth, and newborn health care.

The study intends to listen to voices of the seldom heard groups of sex-workers, those with a disability (or whose newborn is affected), and teen mothers with regards to their experience in pregnancy, childbirth, postnatal care, and newborn care. We too intend to hear from the traditional herbalists or witch doctors and traditional birth attendants and how they influence or would wish to influence maternal and newborn health in this setting.

The details of the population at each socio-ecological level are described in Table 1.

**Table 1:** Study population and inclusion criteria

SEM Level	Description
Individual (Mother) who has had or plans to become pregnant or give childbirth	• Women of reproductive age, regardless of religious identity, socio-economic status, or literacy. • Pregnant and postnatal mothers • Mothers with history of any maternal or newborn morbidity • Mothers with disability (lame, blind, deaf etc) • sex workers, surrogate mothers • Teenage mothers (13–19 years) or even those pregnant before 13 years
Interpersonal (carers) of women with history of pregnancy, childbirth or newborn care	Social networks and social support systems, including • Family (husbands, parents of mothers with any experience of maternal or neonatal morbidity / mortality) • friends, peers, or co-workers of the above individuals. • religious networks (Religious leaders) • customs or traditions (Clan leaders).
Community (wider stakeholders) with interest in maternal and newborn health	Relationships among organizations, institutions, and informational networks within defined boundaries, including • village associations (women’s groups) • community leaders (Local council I-III, religious leaders) • transportation (UTODA leaders, motorcyclist and car drivers) • Village health teams (VHTs) • Traditional healers, witch doctors, Traditional birth attendants
Organizational (Health and social care professionals) interested in maternal and newborn health in eastern Uganda	Organizations or social institutions with rules and regulations for operations that affect how, or how well, maternal and newborn health services are provided to an individual or group; • Schools that include women’s health in the curriculum (primary, secondary). • Tertiary institutions (Universities, colleges) • Health facilities (private and public clinics/ hospitals). Health workers with experience in maternal and newborn health • Community based organisations or groups focused on Women’s Health, REHEMA

##### Inclusion criteria

The individual will be eligible to participate in the PSP if s/he meets any of the 4 broad criteria, aligned to the SEM.

##### Selection, recruitment, and engagement of participants

We will involve participants described in Table 1 in all the key stages of the priority setting exercise. The research team member will select mothers and their families by convenience sampling to participate in this PSP.

We will purposively select participants at each SEM level with current or previous experience in pregnancy or childbirth. We will purposively select vulnerable and marginalised groups including sex workers, women with disabilities, carers of newborns with anomalies and teenagers, traditional herbalists, and traditional birth attendants. The purposive sampling techniques will ensure the representation of voices from vulnerable and marginalised groups of people at each stage of the PSP. A further exponential non-discriminative snowball sampling will be used to sample mothers or families that have experienced any death of the mother or baby or severe morbidity to participate in the PSP.

Recruitment into the PSP will largely be face to face. The research team will physically visit antenatal care clinics, labour suites, postnatal wards, high dependency units, young child clinics, family planning, and immunisation clinics, and gynaecologic wards in community health facilities. They will too visit homes, churches, villages, places of work (shrines, motorcycle stages), and organisations or attend meetings or workshops to recruit participants. Three research members will walk, take motorcycles (Boda-boda) and or taxi to visit each health facility or organisation, meet the institutional representative for introduction and orientation to the staff and clients or participants, will check that the survey launch poster is pinned on the notice boards or offices. In the health facility, the research team will participate in the clinical care of pregnant and postnatal mothers before starting recruitment. Unlike the UK’s online surveys [16, 23–27], these will be face to face surveys. Face-to-face visits are considered to engage participants actively than online surveys [28].

Further, some local members of the Steering group (SG) will identify potential participants and recruit them directly into the study. While representatives of partner organisations will encourage their clients to participate in the prioritisation exercise.

During the face to face recruitment, the research team member will present study information to the participant, obtain informed consent from each participant, and then administer the questions during the surveys.

We will also upload initial and interim prioritisation questions online on the PSP microsite to potentially reach persons with access to the internet in some parts of Mbale. The link to surveys will be sent to WhatsApp groups.

##### Engagement

**Fig. 3:**
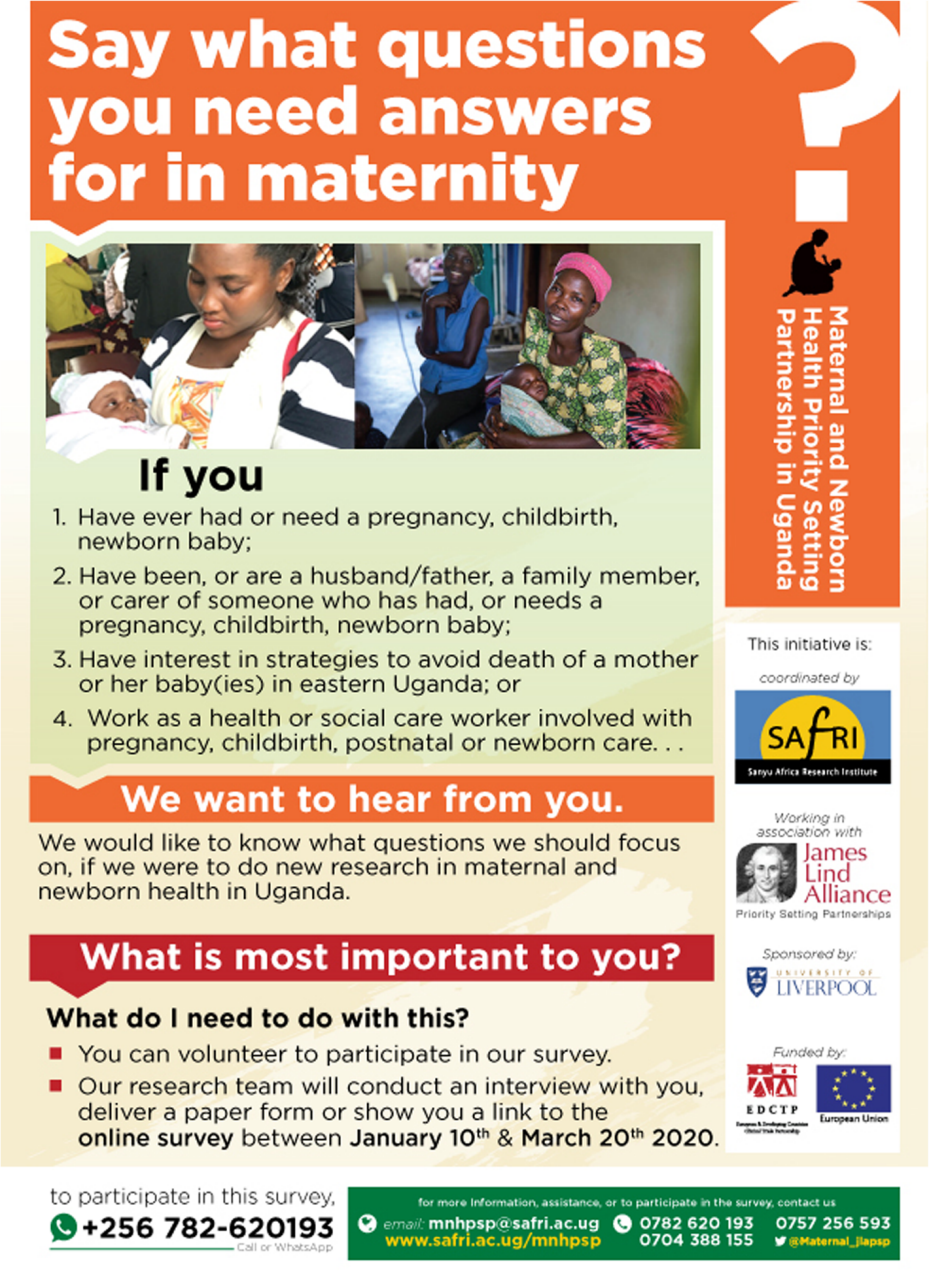
PSP initial launch poster

The research team will directly contact social groups and leaders of institutions, inform and invite them to participate in the PSP. The research team will present the surveys to the partners’ group during the launch meeting and introduction meeting at each organisation or group. The partners’ group will place survey launch poster (Fig. 3), with inclusion criteria and PSP secretariat or research team contacts, in the partners’ premises to advertise the surveys.

The research team will share PSP information to the general public via twitter @maternal_jlapsp and a microsite. The research team together with members of the steering group will hold a launch promotional radio talk show at, *Step Broadcasting media (SBC), a local radio station in Mbale with a coverage of up to 4 million people.*

The members of the steering group and the partners’ group will be encouraged to promote the PSP through their social groups, WhatsApp groups, departmental or village meetings. The research team will create a WhatsApp group to continuously engage and update steering Group members with the PSP recruitment progress.

##### Number of participants by stakeholder group

Table 2 shows the sample size for each phase of the priority setting process.

1. Initial survey

**Table 2:** Sample size for the priority setting partnership

Stage of prioritisation	Sample size (n)	mothers	families	community	health workers	social workers
Initial survey	320	120	40	120	50	10
Interim prioritisation	200	80	20	80	17	3
Final priority setting workshop	27	8	4	6	8	1
Steering group	15					
Lay mothers’ group	5					
Partners group	10					

We will recruit 320 participants in the initial survey. The target sample is 120 (100–140) seldom-heard participants (mothers and their families), 20 (10–30) wider stakeholders, and 80 (60–100) health and social care professionals) for face to face data collection. The survey will also be uploaded online, and we expect to reach about 100 persons with access to the internet. Though this is a small sample size for the initial survey compared to previous JLA PSPs [11, 23–27, 29], we expect at least 300 responses to be generated from this sample. This is within the range of 100 s to 1000s for the initial survey responses from previous PSPs [11].

2. Interim prioritisation

We aim to reach a sample of 200 participants for interim prioritisation. Though this sample size is not based on the average of the previous JLA studies [23–27] for this step of the JLA process, it is above the childhood disability PSPs [11]. Unlike, the previous studies that have used online surveys, this study will involve face-to-face recruitment, which would be costly if we attempted to reach the same target population. However, we shall test the adequacy of the sample size iteratively. The principal investigator will review the results from the 40% data against the next 20% of the data for any significant change in the result.

3. Final priority setting workshop

An average sample size of 27 participants will be invited by the steering group to take part in a one-day workshop. The JLA method recommends a minimum of 12 and a maximum of 30 patients, caregivers, and clinicians [16]. Previous studies have used a sample size of 20 to 43 [23–27].

##### Characteristics of stakeholders

Participants’ characteristics have been described in Additional file 1 and Table 1 for the priority setting surveys. However, for the priority setting workshop, we shall ensure each participant is an ‘expert by lived experience’ about maternal and newborn health. The participants will include, 4 pregnant women, 3 postnatal mothers, 8 carers (husbands, mothers in law), 4 midwives, 2 obstetricians, 2 neonatal nurses, 1 paediatrician, and the rest will be other vulnerable and marginalised population.

##### Support for participants

The research team plans to reimburse participants for travel and time for any PSP meeting away from their homes. We will invite motorcyclists, and sex workers to the research office for data collection and will pay cash for their time and travel.

Mothers involved in priority setting workshops will be encouraged to move with their babysitters who too will receive a modest allowance and a meal. There will be a designated room for breastfeeding mothers during the meetings, but in our settings, these mothers are too allowed to breastfeed freely in the meeting venue.

We will train mothers and other lay members of the public joining the steering group and partners on principles of public involvement, and award them with a certificate of participation at the end of the PSP. We will allow time for the lay public and mothers to offer their voice during the priority setting workshop and any other PSP meeting.

### Identification and collection of research priorities

#### Data collection

##### Initial survey data collection tool

The research group will develop the initial questions iteratively with women, mothers, carers, and professionals in Mbale via individual face to face interviews.

The draft questions will be presented to and reviewed by the partners’ group, and lay mothers’ group in separate half-day group meetings. We will then pilot the questions in Mbale via face to face individual interviews with 10 participants (4-mothers, 2 husbands, 2-midwife, 1-priest, and 1-motorcyclist) to improve the design and clarity of questions.

The Steering Group will review and approve the final initial survey tool (Additional file 3), that will be uploaded onto the open data kit (ODK) (www.opendatakit.org), an electronic data capture system, and loaded onto a mobile smartphone.

The research team will be trained in the protocol, data collection methods and will perform study dry runs before starting data collection.

##### Methods for collecting initial questions

The research team will apply face to face data collection largely through verbal interactions with individuals or groups at a convenient location. In each interview, ‘we will simply ask participants to ask us questions’, which will take approximately 30–60 min to complete. We will begin by asking the participant to tell us their story before the big questions. We will input the participant’s responses directly into the mobile smartphone for most interviews while engaging with the participant. Each interview will be audio recorded on the smartphone with the permission of the participant. Audio records will be transferred to a password-protected computer, labelled with the participant identifier, and used to check against participants’ entered questions.

The research team member will administer printed paper version questionnaires to a small number of participants whenever the mobile phones are off or faulty, complete the form in wet ink, and subsequently enter the paper version responses into the smartphone system.

The research team will conduct individual phone to phone interviews with individuals from either hard to reach areas or due to a busy schedule or transport challenges to access the participants.

The research team will administer the survey questions in the local language understandable to the participant (Luganda, Lumasaba, Lugwere, or Ateso). The research team member fluent in a particular local language will directly translate and ask the questions to the participants who neither speak nor understand English. At the end of the interview, each participant will be asked to confirm their willingness to participate in the interim and priority setting workshop.

The JLA adviser will observe at least two face to face interviews with the participants via the zoom video meeting facility. The student will review each completed form against the audio-record at the end of each field day and the survey.

#### Data management and analysis

##### Data cleaning

The student will export data from the ODK system as a CSV file with the raw questions every two weeks, which will serve as a data handling excel file. We will perform ongoing cleaning of the data while the survey is still open, and an interim sub-analysis of participants’ demographics to identify under-represented study population in the preliminary responses and set a schedule for purposive recruitment.

The student will prepare a complete clean dataset at the end of the initial survey, to categorise and summarise, while maintaining an audit trail. Each participant entry into the data handling excel file will be checked against the consent form, enrolment log and audio recording.

We will double-check each participant’s entries against the audio recording to prevent the research team bias of entering questions based on their technical jargon and losing the lay context. The student or another separate research member will listen to audio recording per participant while comparing with the entered questions to check for consistency of participant’s questions. Any question phrased differently in the data file will be changed to the participant’s phrases in the audio recordings. We will enter the lay questions directly replacing those phrased or entered by the research team member. The cleaning will also involve removing duplicate identification (ID) numbers to participants, assigning new IDs to participants with missing IDs in the dataset.

If the person listening to the audio doesn’t understand the language, then a language expert will be invited to interpret the audio to the research team. We will submit all local names for specific words or herbs during the interviews to a language translator. We also will take pictures of herbs being mentioned in local names by participants and submit them to the research team to confirm the English or botanical name. For the dataset with missing audios, we will contact the participant directly via phone calls to confirm the nature of the questions.

##### Collating and categorising submitted questions

Organise and code the responses.

The student will keep a clean dataset as final on file, share it with the research team and research group, then create a new data file labelled “coded data set”, and will use this to code initial survey responses. The coding process will include removing participant’s initials, names, phone numbers, any blank responses, splitting questions that have multiple sub-questions, giving each question a unique identifier, while maintaining the link to the participant.

The student will code 60% of the participants’ data while each of the two research members will code 20%. The student will review 50% of the data coded by research members while each research member will review 30% of the data coded by the student.

Identify out-of-scope questions.

The student with the research team members will continue to code the dataset. This will involve creating a column in the dataset for in-scope or out-of-scope with its reason and checking each question against the agreed PSP scope, as defined earlier in this protocol. The research team will hold a zoom meeting with the JLA adviser and the academic research group to check the exact interpretation of the scope.

We will analyse out-of-scope questions separately from the in-scope questions and generate tables for review by academic research and steering group members. The research team will prepare frequently asked questions (FAQ) document for the questions of information and advice, which will be shared with antenatal care units, health facilities, district health offices, and the ministry of health.

Sort into themes and summarise in-scope questions.

The research team will sort out all in-scope questions into overall original themes, namely; pregnancy and its complications, labour and its complications, diagnosis, prevention, and treatment.

The student will export the in-scope questions or data into NVivo 10 software, to generate emerging themes following the principles of the thematic content approach for analysis [30]. Another research member will peer-review the reliability of emerging themes and issues regularly; (for every 20 participants, will review 1 participant by manually generating emerging themes). They will swap a portion of their respective data and compare findings for consistency. Any discrepancies or issues arising from specific responses will be adjudicated by a member of the academic research group or JLA adviser and discussed by the steering group if necessary. The research team will share the list of emerging themes with the academic research group and steering group to review, confirm, and approve.

The research team will code questions asked only once as single questions with the emerging theme. However, they will group similar in-scope questions, which are asked at least twice, to form an indicative question representing all these questions in terms of the language and context.

Discuss and agree on questions with the steering group.

The research team will present the list of indicative questions to the lay mothers’ group in a 3-h face to face meeting at a central place and check whether they agree with how the questions have been categorised and summarised.

The research team will organise a steering group meeting, in which members will review, comment, and confirm themes, indicative in-scope questions, single in-scope questions, and out-of-scope questions.

##### Finalise the long list of questions for the interim prioritisation

Evidence check for research questions.

The student will check each indicative question against existing literature or systematic reviews through a rapid review of the literature. An independent librarian at Busitema University in Uganda will do a separate literature search using a predefined systematic criterion and the search results will be shared with the student to double-check the search results. Any discrepancies will be resolved by another search by the librarian at the University of Liverpool. We will apply the GRADE system for rating the quality of a body of evidence as a guideline wherever evidence is not clear [31]. The student will search for systematic reviews relating to maternal and newborn health research priorities, search for clinical guidelines for pregnancy, childbirth, and neonatal care in Uganda and related countries, search for articles on topics raised by the questions.

The research team will perform the following for any answered question;

Provide summary evidence against each question, including s as appropriate,

Review all the questions with the steering group.

Following the evidence review, the steering group will agree which questions are to be removed as already being answered.

Where questions are partially answered by research, they will be amended to include the un-answered part of the question.

The remaining indicative questions and any single questions as agreed by the steering group will form the final long list of unanswered questions by research.

Number of unanswered research questions.

The research team will group questions for maternal health alone, newborn health alone, and later combine maternal and newborn health to form three separate long lists of questions.

A one-off face-to-face steering group meeting will be held to discuss the three long lists of questions and agree on the number of questions on each list to go forward for interim prioritisation. In this PSP, we will consider up to 70 questions for maternal health alone, 70 for newborn health alone, and 70 for both maternal and newborn health together. Previous PSPs have considered an average of 65 (range 40–114) research questions on the long list.

The following criteria will be used to reduce the large list of indicative questions to the proposed number.

The question based on a submission by utmost two participants will be prioritised lower than questions from several participants in the initial survey.

The question based on submission from one socio-ecological model level or group will be prioritised lower than questions from more SEM levels.

The removed questions will be checked to ensure that questions known to be coming from ‘seldom heard’ persons are retained.

### Prioritisation of research topics/ questions

#### Interim prioritisation

##### Methods and criteria

The research team will conduct face to face data collection largely through verbal interactions with each participant at each SEM level.

The research team will arrange the three agreed longlist of questions separately in a better format for visualisation on the paper version or the mobile smartphone.

The research team will administer the questions to individual participants in a chronological order starting with the longlist for both maternal and newborn health combined, followed by a long list for maternal health alone, and will end with the longlist for newborn health alone. There will be a resting period between the second and last long list.

##### Ranking process

Each participant will read or be read the long list of questions in random order from the phone or the paper version. The research team member will then ask each participant to choose any 10 most important questions on each of the three-long list of questions separately and have the responses entered into the questionnaire, following the JLA approach.

Based on the experience of the JLA adviser, asking people to rank their top 10 questions does not add much to the value of the data, hence they will only be asked to choose and not rank. There are examples of PSPs that have applied this ranking approach [11].

In the process, ‘we will simply ask each participant to choose her/his most important questions’, which will take approximately 90 min to complete.

We will hold three steering group meetings to discuss and agree on an overall shortlist of 20–30 top questions from each longlist to be taken forward to the final priority setting workshops.

This is planned to take 12 weeks.

#### Final prioritisation workshop

##### Method and criteria

This will be a face-to-face group priority setting workshop using participatory mixed methods. The research team will hold three priority setting workshops on three separate dates with three separate groups of participants. The participants will have similar characteristics across the three groups.

The first priority setting workshop will be for the combined maternal and newborn health priorities, while the second priority setting workshop will be for the maternal health questions alone, and the third priority setting workshop will be for the newborn health questions alone.

##### Procedure

Each priority setting workshop will occur in a central meeting place for all participants. We will follow the standard JLA approach. The process will encourage open discussion and involvement of all group members guided by an independent JLA chair and assisted by the research team and observed by the academic research group.

The ranking exercises will be based on nominal group techniques [15].

All participants will be made aware of the purpose of the workshop at the beginning, to generate the top 10 research priorities representing the views of all those who participated. The workshop will begin with small-group debate and discussion to challenge and explore the final questions on the shortlist before the final plenary ranking session.

The JLA adviser will facilitate this process and ensure transparency, accountability, and fairness and ensuring that the views of the vulnerable and marginalised population are considered. Participants will be expected to declare their interests in advance of this meeting.

#### Status of the study

The present study commenced in January 2020. Participants are currently being recruited to the study. The outbreak of the COVID-19 pandemic and the lockdown restrictions in Uganda subsequently affected our timelines. Data collection for the initial survey will be completed once the COVID-19 lockdown restrictions are lifted, possibly in October 2020. Recruitment for the interim prioritisation will be finalised in May 2021 and priority setting workshops in August 2021.

#### Output

We will generate research topics, themes, or areas and questions and compare how people chose maternal health questions over newborn health questions in this setting. The original research questions for all the three long lists will be maintained non-technical to avoid missing context and value around these questions from the non-researchers.

The Academic research group plans to translate the questions into technical focussed research questions with a specific structure, i.e. the Population, Intervention, Comparator, Outcome (PICO) format.

### Evaluation and feedback

#### Patient and public involvement (PPI)

For purposes of reporting, Table 3 summarises some of the levels and methods of public involvement planned in this PSP.

**Table 3:** Levels and methods of public involvement in maternal and Newborn health PSP in Uganda

Stage of JLA process	Public involvement Level	Public involvement method	Specific involvement activities
1. Initial enquiry	None	N/A	N/A
2. Form Steering group	consult	Interviews	Invitations
3. Identify and invite partners	collaborate	Representatives of mothers, health workers	Recruit wider stakeholders
4. Inaugural steering group meeting	collaborate lead/ support	PSP SG group Academic research group	Meetings Teleconferences
5. Initial launch meeting	collaborate lead/ support	Partners’ group meeting/ Workshop	Stakeholders workshop Pilot testing the data tool Comment on questions Launch initial survey
6. Initial survey to gather questions	consult Involve Lead/support	Individual interviews Focus groups Lay mothers’ review SG reviews	e-data capture (mobile) Paper based survey SG and lay mothers’ Meetings
7. Data processing & refining questions	User controlled research Lead/support	SG review Academic research group	Meetings Phone calls, WhatsApp Emails Zoom
8. Verifying indicative questions	User controlled research Lead/support	SG review Academic research group	Meetings Phone calls, WhatsApp Emails Zoom
9. Presentation of raw submissions for interim prioritisation	User controlled research	SG review	Meetings Phone calls, WhatsApp Emails Zoom
10. Interim prioritisation	consult Involve Lead/support	Face to face survey Focus groups Lay mothers’ review SG reviews	e-data capture (mobile) Paper based survey
11. Identify top 30 Analysis	Collaboration	Steering group review	Face to face meeting Zoom
12. Final priority setting Identify top 10	Collaborate	Workshop in Mbale	Small groups Plenary sessions
13. Next steps	Collaborate	Dissemination Quality assurance	Checking data
14. Communication	User controlled Lead/support	Dissemination Conferences, twitter	stakeholders meeting Preparation of newspaper article

#### Evaluation of the patient and public involvement

The research team will track and report on PPI processes within this research including the experiences and impact of public involvement from the perspectives of the steering group members, partners, and participants.

We will use an activity log to record PPI outcomes during any meeting or workshop (Additional file 2). Each meeting member will complete an evaluation form embedded in the impact log at the end of each meeting.

Every participant in both the initial and interim prioritisation will have their satisfaction assessed about the process of involving them to identify and choose questions respectively. During the final priority setting workshop, participants will complete an evaluation form.

Comparative analyses of the responses from participants will also be performed to determine the impact of different types of participants on proposed research questions and establish how the maternal research priorities are chosen over newborn health research questions.

JLA will undertake anonymous evaluation surveys for the academic research group, the research team, the steering group members, and the final priority setting workshop participants.

#### Feedback and PSP dissemination

The research team will organise local dissemination meeting at each partner’s site and present the results to the staff. A list of top 10 questions for maternal and newborn health combined, maternal health alone, or newborn health alone will be printed on a paper for pinning in each partner’s premises. The partners will be encouraged to present results to their clients.

The research team will organise radio and TV talk shows in Mbale and share results with the general public in the local languages.

The protocol and results of the MNH-UG PSP will be disseminated through the PSP website, the Sanyu Africa Research Institute website, twitter handle, WhatsApp and Facebook, peer-reviewed publications, academic conferences, and through formal presentations to the stakeholders. The JLA will also capture and publicise the study protocol, key events, and results on its website.

### Funding and conflict of interest

The PSP is financially supported by the EDCTP through a grant to the BabyGel cluster randomised trial **(ref: RIA2017MC-2029-BabyGel)**. EDCTP has no part in the execution of the study and write-up. The James Lind Alliance provided partial contribution towards the JLA adviser’s time. The University of Liverpool offered a Ph.D. tuition waiver for the student while the Sanyu Africa Research Institute paid the student’s stipend during the project period. The budget for the PSP project alone is estimated at €28,000.

All steering group members and priority setting workshop members will declare their interests at the start of the project and workshop respectively.

## Discussion

The maternal and newborn PSP in Uganda is translating the JLA principles not just into the local language, but into the local setting. The current protocol elaborates JLA methods for use with a new topic and in a new setting. This PSP challenges the usual process of PSPs in western settings, where participants are generally literate and informed of their condition, where there are existing support and professional networks for the condition, and digital technologies are in common use. We anticipate the findings could be rapidly incorporated into the future World Health Organization and other international research priorities.

This study will, for the first time in rural Uganda, provide new learning for ensuring the voices of seldom heard and under-represented complex populations in rural villages and health facilities are heard to form the research agenda in pregnancy, childbirth, and newborn health.

The PSP provides the steps that necessary for an academic research group to follow while setting up a priority setting partnership for purely academic training purposes at postgraduate level.

Unlike other PSPs, this one will adopt a face to face collection of questions from the participants. It will be a non-internet-based approach; there is hardly any internet access in the villages of Uganda and any efforts to use the internet to collect questions from the lay public would not yield the locally appropriate responses. Further, the Corona virus disease 2019 pandemic creates a new challenge. We therefore will demonstrate the feasibility of using phone calls to consent and collect raw questions from participants in this setting while adhering to the COVID-19 national guidelines for conduct of research in Uganda [32].

Unique with this PSP is the formation of a sub-group of lay mothers as part of the steering group. This is a group of mothers who are not English speaking and will represent views of the majority of the rural women who will be participating in the surveys.

The steering group agreed on a broad remit for this PSP of maternal and newborn health. The scope is further subdivided to explicitly include first and third trimesters This aims at exploring the critical questions during this period, especially in rural Uganda where first-time mothers are exposed to traditional practices with unknown effectiveness. We too included the immediate postpartum period due to the high risk for maternal and neonatal mortality during this period [33, 34].

The study will inform the establishment of a protocol for research prioritisation of Maternal and Newborn Health (MNH) in Uganda. If proven effective and appropriate in MNH, this methodology will ultimately be adopted as one of the methodologies in identifying research priorities for other disease conditions in Uganda or any LMICs.

The results of this study will inform the design of studies out of women’s voices or seldom heard population. The study findings will guide the Ministry of Health Uganda and current funders in resource allocation, as well as the Royal College of Obstetricians and Gynaecologists (RCOG) global health division and other researchers in the research agenda development. We anticipate the findings could be rapidly incorporated into the future WHO research priorities and or prioritisation exercise (facilitated by the University of Liverpool Department of Women’s and Children’s health, WHO Collaborating Centre status).

## Conclusions

The Maternal and Newborn Health Priority Setting Partnership in Uganda will, for the first time, identify the most pressing unanswered questions about the pregnancy, childbirth, and after childbirth in under-served populations in villages and health facilities within the rural settings of Uganda. This will ensure that future research can be prioritized according to the local needs of mothers, carers, families, and professionals with interest in strategies to avoid maternal and newborn morbidities and mortalities.

## Supplementary information

**Additional file 1.** Characteristics of governance team and participants.

**Additional file 2.** PPI Activity log for recording JLA PSP Meetings.

**Additional file 3.** Initial survey data tool.

## Data Availability

Data will be published in an appropriate data repository and also freely available upon request from the principal investigator.

## Abbreviations

JLA: James Lind Alliance
LIR: Low income regions
MNH: Maternal and Newborn Health
PSP: Priority Setting Partnership
RCOG: Royal College of Obstetricians and Gynaecologists
SSA: sub-Saharan Africa
SG: Steering group
