# Supplementary material for: Maternal and newborn health priority setting partnership in rural Uganda in association with the James Lind Alliance: a study protocol
Source: Res Involv Engagem. 2020 Sep 22;6:57. doi: 10.1186/s40900-020-00231-4 (PMC7506205; doi:10.1186/s40900-020-00231-4)
Supplement: Supplementary file 2 — Additional file 2. PPI Activity log for recording JLA PSP Meetings. [file 40900_2020_231_MOESM2_ESM.docx]

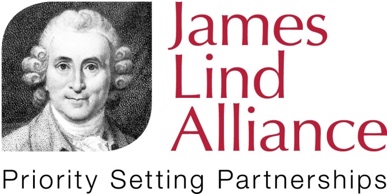

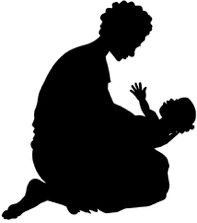


Maternal and Newborn Health

Priority Setting Partnership in Uganda

Additional file 2: PPI Activity log for recording JLA PSP Meeting outcomes

| Date | Type of event/ activity | Participants (attendees/apologies) | Meeting objectives or purpose | What did we do? What was said/ suggested/ recommended | What was changed? | Impact (positive & negative), Other comments |
| --- | --- | --- | --- | --- | --- | --- |
| 18-12-2019 | Inaugural steering group meeting | *All steering group members* | *The research team sent out an invitation pack to all proposed members of the steering group. This included formal appointment letter, invitation letter, agenda or programme, protocol, steering group terms of reference, declaration of interests and privacy form, and PSP poster.*  *The meeting objectives included:*  To introduce Steering Group members to the JLA PSP process  To present the plan for the Maternal and Newborn Health PSP in Uganda  To initiate discussion, answer questions and address concerns related to the protocol, initial survey tool  To discuss and agree on the scope of the Maternal and Newborn Health Priority Setting Partnership in Uganda and ways to maximise the responses to the process  To identify additional partners which may commit to the PSP and confirm individuals who will be those organisations’ representatives  To establish principles upon which an open, inclusive, and transparent mechanism can be based on contributing to, reporting, and recording the work and progress of the PSP in this specific setting. | *SG member suggested and approved study setting* |  | *Topic guide flowed better, and I was not uncomfortable asking the question during the interview* |
|  |  |  |  | *SG proposed to include the following groups to the study population; women with disability, traditional herbalist and witch doctors, special mothers like sex workers, administrators, ambulance drivers, and cyclists.* | *Added the following to the study population; women with disability, traditional herbalist and witch doctors, special mothers like sex workers, administrators, ambulance drivers, and cyclists.* | *Inclusion of vulnerable and marginalized population in the study* |
|  |  |  |  | *SG recommended extending the newborn health period from 28 days to 6 weeks, to align to the postnatal period for the mother.* | *The scope was changed from*  *from 28 days to 6 weeks* | *This will match the newborn period with the postnatal period for maternal health* |
|  |  |  |  | *SG composition unanimously agreed to the principles of JLA and their roles.*  *Each member completed the declaration of interests and privacy form and took a photo for profile* |  |  |
|  |  |  |  | *SG recommended forming the PSP WhatsApp group and a basic website.*  *One SG member offered radio airtime to disseminate the survey* | *PSP WhatsApp group formed.*  *Monthly radio talk shows to be held on Abakyala Twogere* | *This will reach many people in areas with radios alone.* |
|  |  |  |  | *SG approved the formation of the lay mothers’ group and its subsequent meetings* | *Research team to recruit Members to the lay mothers’ group, train and organize a meeting* | *Lay mothers’ group will ensure the voice of the marginalized and disadvantaged women is reflected in all decisions* |
|  |  |  |  | *SG approved proposed partners and suggested to invite new partners to the partners' group (Kizito Babies Home, Mt Elgon Hospital, Budaka District Health office, Mbale DLG)* | *New partners were invited to the partners' group list and the partners' awareness meeting held* |  |
|  |  |  |  |  |  |  |

| Date | Type of event/ activity | Participants (attendees/apologies) | Meeting objectives or purpose | What did we do? What was said/ suggested/ recommended | What was changed? | Impact (positive & negative), Other comments |
| --- | --- | --- | --- | --- | --- | --- |
| 10-01-2020 | Partners group awareness and an initial survey launch meeting | *All partners.*  *The research team contacted the proposed partners via phone, email, and face to face.*  *Those who confirmed were sent appointment letters and invited to the partners or launch meeting with the day’s agenda.*  *11 Out of 15 partners attended the launch meeting* | *The meeting objectives included:*  To welcome and confirm members of the partners' group to the JLA PSP  To present the plan for the Maternal and Newborn Health PSP in Uganda  To initiate discussion, answer questions, and address concerns related to the PSP information, initial survey tool, and poster.  To discuss and agree on the strategies for recruiting participants from the partners.  To confirm individuals who will be partners’ representatives and the principal contacts  To launch the initial survey.  Each partner was given a partner’s information pack with the following;  PSP information leaflet, launch poster, appointment letter, the day’s presentations | *Partners’ group representatives suggested the inclusion of new partners, namely Spotlight Africa, Budaka District health office, CURE Children’s hospital, Nzu Ya Masaba.* | *Added the new partners, and shared with the SG for approval and confirmation* | *New partners with a diverse patient characteristic were included to contribute to the study population* |
|  |  |  |  | *Members introduced each other by name, occupation, the institution of affiliation, and their unique experience with pregnancy, childbirth, and newborn health.*  *In memory of Linda, this PSP was emphasized following her death on 09/01/2020 immediately after giving birth in labour suite of Mbale RRH.*  *Introduced the PSP to the partners and the proposed recruitment strategy. The partners suggested and recommended to pin the PSP launch poster in their institutions, to hold meetings with all staff to engage them about the PSP.* | *The principal contacts from each partner organization were confirmed and all contact details were provided.*  *Each partner was given a launch poster for pinning in their institution’s notice boards.* | *Easy to contact the partner and be introduced into the facility for recruitment of participants* |
|  |  |  |  | *Partners completed the Partners form to confirm their interest in the PSP.* |  | *Their commitment to the PSP is important in recruitment* |
|  |  |  |  | *The research team presented the press release to partners, titled “mother’s voice matters in pregnancy and childbirth”, which was approved and forwarded to the media practitioners.*  *The Hospital Director, Mbale RRH then launched the initial survey officially* | *Press released for the PSP launch.*  *Each partner was given the launch poster (Figure 3) for promotion of the initial survey in his/her organization* | *Research team ready to start recruitment into the initial survey* |
|  |  |  |  | *SG suggested a radio talk show programme for the PSP to include; medical points, ten key messages, to avoid medical jargon, take-home messages for the audience* | *Radio Talk show dubbed Abakyala Twogere scheduled* | *Will make the public aware of the PSP, address concerns in the PSP and disseminate results* |
| 31-01-2020 | Lay mothers’ group meeting | *One SG member identified 10 lay mothers, who were invited to a meeting in Mbale by the research team. Only 5 mothers turned up and were confirmed to compose the lay mothers’ group.* | *Meeting objectives include*  *To train mothers about principles of public involvement and the PSP.*  *To seek the input of lay mothers about the sensitivity of questions in the data collection tool and the recruitment process* | *The mothers suggested that the research team member should have a section in the questionnaire where they first hear or listen to the participant’s story before going to ask him or her to tell the big questions.*  *They suggested that the research team should move to the homes to recruit participants beginning afternoon as many people will be digging in the morning.*  *The group discussed about the preference for male or female research team or recruiters. They felt that presence of male but more preference towards females.* | *We added a general response section to the initial survey questionnaire to capture the participant’s story before exploring or asking for big questions.*  *The research team will visit homes in the afternoon and early evenings*  *Three research assistants were female with one male who is the student* | *Allowing the participant to first tell their story creates a better understanding of the context and nature of questions proposed by the participants* |
