# Supplementary material for: Maternal and newborn health priority setting partnership in rural Uganda in association with the James Lind Alliance: a study protocol
Source: Res Involv Engagem. 2020 Sep 22;6:57. doi: 10.1186/s40900-020-00231-4 (PMC7506205; doi:10.1186/s40900-020-00231-4)
Supplement: Supplementary file 3 — Additional file 3. Initial survey data tool. [file 40900_2020_231_MOESM3_ESM.docx]

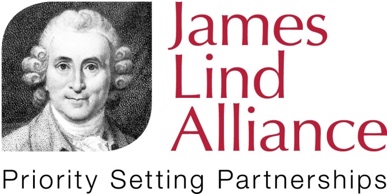

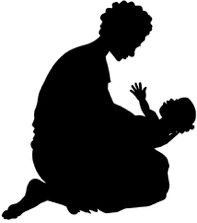
Maternal and Newborn Health Priority Setting Partnership in Uganda

***Do you have any questions about pregnancy, labour or childbirth and newborn health that you seek and or have been unable to find answers for?*** This study aims to prioritise the most important research questions for maternity and newborn health in Uganda from a community perspective. We especially want to consider the opinions of ‘*experts by experience*’ in under- represented population. To do this, we will interview pregnant and postnatal mothers, their carers, family members, health and social care professionals as well as the wider community. Particularly, we want to hear from those who have suffered complications or difficulties during pregnancy or childbirth or their newborns had adverse outcomes, so that we can design research around their needs.

We, hence, would like to invite you to take part in this study. You will suggest any questions about the prevention, treatment, diagnosis and care during pregnancy, childbirth, after childbirth and care of their newborns that you seek and or have been unable to find answers for.

By taking part in this study, you are agreeing to allow us to publish the questions you identify. Your name or organisations will not be published.

**What do we mean by “unanswered questions”?**

We are looking for questions about the prevention, diagnosis, treatment and care of pregnant and postnatal mothers and or their newborns that are currently not answered by research evidence. Here are some examples of unanswered questions from mothers and health workers:

Mothers

- *Is it necessary for all women to take iron, folate, or vitamins routinely during pregnancy?*
- *What is the safest effective way of controlling nausea in early pregnancy?*

Health workers

- *Why do most women hardly turn up for antenatal care in the first 3 months of pregnancy, and how can it be improved?*
- *Does adding steroids to local anesthetics improve their effectiveness in relieving perineal pain?*
- *Do postnatal pelvic floor exercises reduce the frequency of stress incontinence postpartum?*

What unanswered questions do you have about the **pregnancy, childbirth, after childbirth, and the newborn baby**? *Please fill in any that you have in the correct box or as entered in by Research assistant (You can submit as many or as few as you like)*

SECTION I: Please tell us your big questions:

# **GENERAL SECTION**

_________________________________________________________________________________________________________________________________________________________________________________________________________________________________________________________________________________________________________________________________________________________

What question(s) about the **Pregnancy in the first three months| Antenatal care** would you like to see answered by research?

# **MY FIRST QUESTION**

_________________________________________________________________________________________________________________________________________________________________________________________________________________________________________________________________________________________________________________________________________________________

# **MY SECOND QUESTION**

_________________________________________________________________________________________________________________________________________________________________________________________________________________________________________________________________________________________________________________________________________________________

What question(s) about the **Pregnancy in the last three months| Antenatal care** would you like to see answered by research?

# **MY FIRST QUESTION**

_________________________________________________________________________________________________________________________________________________________________________________________________________________________________________________________________________________________________________________________________________________________

# **MY SECOND QUESTION**

_________________________________________________________________________________________________________________________________________________________________________________________________________________________________________________________________________________________________________________________________________________________

What question(s) about the **treatment of complications during pregnancy** would you like to see answered by research?

# **MY QUESTION**

_________________________________________________________________________________________________________________________________________________________________________________________________________________________________________________________________________________________________________________________________________________________

What question(s) about the **Labour| childbirth** would you like to see answered by research?

# **MY FIRST QUESTION**

_________________________________________________________________________________________________________________________________________________________________________________________________________________________________________________________________________________________________________________________________________________________

# **MY SECOND QUESTION**

_________________________________________________________________________________________________________________________________________________________________________________________________________________________________________________________________________________________________________________________________________________________

What question(s) about the **treatment of complications at childbirth** would you like to see answered by research?

# **MY FIRST QUESTION**

_________________________________________________________________________________________________________________________________________________________________________________________________________________________________________________________________________________________________________________________________________________________

# **MY SECOND QUESTION**

_________________________________________________________________________________________________________________________________________________________________________________________________________________________________________________________________________________________________________________________________________________________

What question(s) about **Postnatal care| for the mother in the first 6 weeks after childbirth** would you like to see answered by research?

# **MY FIRST QUESTION**

_________________________________________________________________________________________________________________________________________________________________________________________________________________________________________________________________________________________________________________________________________________________

# **MY SECOND QUESTION**

_________________________________________________________________________________________________________________________________________________________________________________________________________________________________________________________________________________________________________________________________________________________

What question(s) about **Newborn care in the first one week of life** would you like to see answered by research?

# **MY FIRST QUESTION**

_________________________________________________________________________________________________________________________________________________________________________________________________________________________________________________________________________________________________________________________________________________________

# **MY SECOND QUESTION**

_________________________________________________________________________________________________________________________________________________________________________________________________________________________________________________________________________________________________________________________________________________________

What question(s) about **Newborn care in the first one month** would you like to see answered by research?

# **MY FIRST QUESTION**

_________________________________________________________________________________________________________________________________________________________________________________________________________________________________________________________________________________________________________________________________________________________

# **MY SECOND QUESTION**

_________________________________________________________________________________________________________________________________________________________________________________________________________________________________________________________________________________________________________________________________________________________

# Any MORE questions you would like to tell us?

_________________________________________________________________________________________________________________________________________________________________________________________________________________________________________________________________________________________________________________________________________________________

On a scale of 1- 5, how do you rate your satisfaction towards saying what questions you need answers for in maternal and Newborn health? (**1-**less satisfied **5-** most satisfied)

_________________________________________________________________________________________________________________________________________________________________________________________________________________________________________________________________________________________________________________________________________________________

SECTION II: Please tell us a little bit about yourself:

**Which of the following best describes you?**

□  A pregnant mother

□  A postnatal mother

□  A mother with previous severe morbidity from pregnancy or childbirth

□  A mother or carer of a newborn baby with previous severe morbidity

□  A carer or family member of a mother with previous pregnancy

□  A carer or family member of a mother with previous severe morbidity from pregnancy or childbirth

□  A health worker, specify…………………

□  Others, specify………………………………………..

**Are you**

□ Male
□ Female

□  Prefer not to say

**What is your age?**

□  Under 15

□  15-35

□  36-45

□  46-55

□  56 and above

□  Prefer not to say

**What is your highest level of education?**

□  No formal education

□  primary education, specify highest class attended

□  secondary education, specify highest class attended

□  college education, certificate/diploma

□  University education- Degree, master, PhD

□  Any other, please specify…………………….

□  Prefer not to say

**What is your primary occupation?**

□  Peasant farmer

□  Teacher

□  social worker

□  unemployed

□  Any other, please specify…………………….

**Would you like to help us in the next stage of deciding the top 10 research questions? If Yes, please provide the details below**

□  Name: _______________________ □  Phone: _____________________________

□  GPS coordinates:____________ □  village: _____________________________


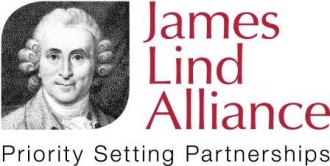

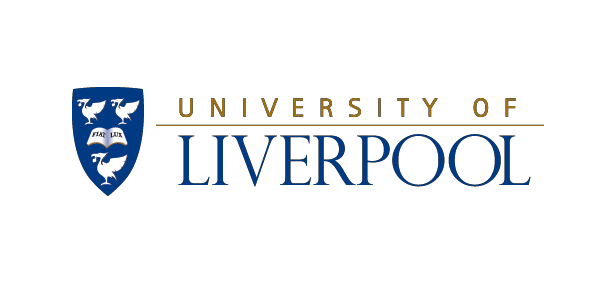

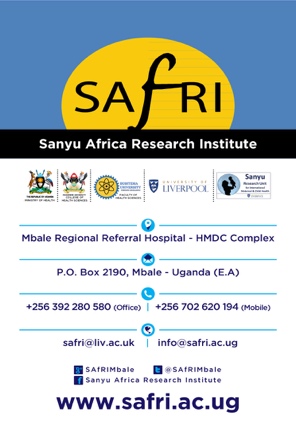
website: [www.safri.ac.ug/mnhpsp](http://www.safri.ac.ug/mnhpsp)

Name of data collector: _____________ Signature: ____________
